# Comparative evaluation of TK SLC-L, a rapid liquid mycobacterial culture medium, with the MGIT system

**DOI:** 10.1186/1471-2334-14-130

**Published:** 2014-03-06

**Authors:** İhsan Hakkı Çiftci, Engin Karakeçe

**Affiliations:** 1 Department of Microbiology, School of Medicine, Sakarya University, Sakarya, Turkey

## Abstract

**Background:**

The present study was conducted to assess the efficiency of using TK SLC-L (Salubris, Inc.) for the primary isolation of mycobacteria from clinical samples by comparing it to the MGIT detection system (Becton Dickinson Diagnostic Instrument Systems). Although TK SLC, a biphasic medium, has been evaluated previously, this is the first study to evaluate TK SLC-L, a liquid medium.

**Methods:**

Clinical specimens from a total of 146 clinically suspected cases of tuberculosis were studied. Each processed sample was evaluated by ZN staining and inoculated into TK SLC-L and MGIT tubes. The TK SLC tubes were incubated in a MYCOLOR TK while the MGIT tubes were incubated in a MGIT system. Growth, indicated by automated systems, was confirmed through production of a smear and microscopic evaluation after ZN staining.

**Results:**

Mycobacterial growth was positive in 35 TK SLC-L and in 34 MGIT samples. Although the growth detection time was approximately 3 to 5 days shorter, on average, with the MGIT system, the contamination rate was significantly lower using TK SLC-L. The total time spent for the repetition of cultures for contaminated samples in MGIT make the total return time for culture results equal to or longer than the time required by TK SLC-L.

**Conclusions:**

The TK Culture System using TK SLC-L is an efficient system and possible alternative to other rapid mycobacterial culture systems.

## Background

Given its global prevalence, devastating morbidity, and massive mortality, tuberculosis (TB) has been among the most important of human infections for many centuries. In recent years, the prevention, diagnosis, and treatment of TB have become more complicated because of changing epidemics [1]. The rapid diagnosis of *Mycobacterium tuberculosis* is essential to implement adequate antimicrobial therapy and effective disease control. Culture is the gold standard method for the diagnosis of TB. In particular, liquid automated culture systems, which offer high sensitivity for the early diagnosis of TB, are important tools. Middlebrook broth is a commonly used medium in the rapid mycobacterial culture system MGIT (Becton Dickinson Diagnostic Instrument Systems, Towson, MD, USA) and in other systems such as the BacT ALERT 3D (bioMérieux, Marcy l’Étoile, France) and ESP Culture System II (Trek Diagnostics, Westlake, OH, USA); however, these systems require the addition of oleic acid, albumin, dextrose, catalase, and selective antimicrobials before inoculation of the processed sample. These extra manipulations increase the risk of contamination [2,3].

TK Media have the advantage of being ready-to-use. TK SLC-L (Salubris, Inc., Boston, MA, USA) includes all of the chemicals required for growth plus five selective antimicrobials: polymixin B, piperacillin, amphotericin B, nalidixic acid, and trimethoprim. TK Media indicate mycobacterial growth by changing colour from red to yellow. If contaminants grow in the medium, it turns to green enabling the differentiation of mycobacterial growth from contamination. Growth in TK Media can be followed visually or with an automated incubator reader, the MYCOLOR TK [4]. Cords formed by *M. tuberculosis* can be seen in smears made from culture tubes, indicating mycobacterial growth. Although TK SLC, a biphasic medium, was evaluated previously, this is the first study to evaluate TK SLC-L, a liquid medium. This study was conducted to assess the efficiency of using TK SLC-L by comparing it to MGIT for the primary isolation of mycobacteria from clinical samples.

## Methods

A total of 146 clinical samples from 146 suspected TB patients were processed at Sakarya Education and Research Hospital (Sakarya, Turkey) over a three-month period (October-December 2012). This is an experimental study. In this study, *in vitro* samples were investigated prospectively. Extra samples were not taken from any patient.

All samples included in this study were sputum. The samples were decontaminated by the NaOH-NALC decontamination/concentration method (Kubica, 1963) using a ready-to-use kit, MYCOPROSAFE (Salubris, Inc.) [5]. The duration of decontamination was 15 min. Before inoculation to culture media, each sample was examined by microscopy for the presence of acid-fast bacilli (AFB) using Ziehl-Neelsen (ZN) staining.

Each processed sample was inoculated into TK SLC-L and MGIT tubes. The TK SLC-L tubes were incubated at 37ºC in a MYCOLOR TK while the MGIT tubes were incubated in a MGIT system. Growth was monitored by the instruments for 6-8 weeks. Positive cultures were confirmed by preparing a smear for microscopic evaluation after ZN staining. Contamination was confirmed by microscopic evaluation and conventional culture on 5% sheep blood agar. Samples that did not show growth in the automated instruments at the end of the incubation period were reported as negative, after confirmation of the absence of AFB by microscopy.

All data were analysed using SPSS (version 17.0); frequency, cross-tab, Pearson’s correlation, and McNemar tests were used. These analyses were selected to evaluate the degrees of association and strengths between the tests. Sensitivity, specificity, positive predictive value, negative predictive value, and accuracy were calculated according to Bayes theorem [6].

## Results

Among the 146 samples included in this study, 11 (7.5%) were acid-fast bacillus-positive by microscopic examination. There was no growth in either system from 100 (68.5%) samples. From a total of 46 (31.5%) samples, mycobacteria were isolated in at least one type of system. The TK SLC-L and MGIT systems showed a similar percentage of culture positivity (24.0 and 23.3%, respectively). The number of mycobacteria isolated using TK SLC-L was higher than that using MGIT; however, the difference was not statistically significant. Contamination of the cultures by other organisms was observed in 2 (1.3%) TK SLC-L tubes and 20 (13.7%) MGIT tubes. The difference was statistically significant, as evaluated by the McNemar test (p < 0.001). All contaminating organisms were detected by microscopic examination and conventional culture.

The time to growth detection varied between 5.2 and 34.8 days (mean, 18.3 days) in TK SLC-L and between 2.0 and 34.1 days (mean, 13.1 days) in MGIT. The median time to growth detection was 15.1 (SD, ± 9.4) days for TK SLC-L and 7.7 (SD, ± 8.7) days for MGIT. There was no statistically significant difference between the mean growth detection times.

When the evaluated test results were compared to total culture positivity, the sensitivity of microscopy, TK SLC-L, and MGIT was 23.9, 76.1, and 73.9%, respectively. The positive and negative predictive values were 100 and 89.2% for TK SCL-L and 100 and 88.0% for MGIT, respectively. The correlation values between total culture positivity and the evaluated tests for microscopy, TK SLC-L, and MGIT were 42.1, 80.6, and 53.0%, respectively. These and additional results are summarised in Table 1.

**Table 1:** Comparison of microscopy, MGIT, and TK SLC-L for the detection of mycobacteria from clinical samples

**Variables**	**Microscopy**		**TK SLC-L**		**MGIT**	
	**n**	**%**	**n**	**%**	**n**	**%**
Total positive results among the 146 samples	11	7.5	35	24.0	34	23.3
Among 46 culture-positive samples	11	23.9	35	76.1	34	73.9
Contamination	NA	NA	2	1.3	20	13.7
Min/max duration for growth detection (days)	NA	NA	5.2/34.8		2.0/34.1	
Mean time to growth detection (days)	NA	NA	18.3		13.1	
Median time to growth detection (days)	NA	NA	15.1		7.7	
Sensitivity* (%)	23.9		76.1		73.9	
Specificity* (%)	100		100		100	
Positive predictive value* (%)	100		100		100	
Negative predictive value* (%)	74.1		89.3		88.0	
Accuracy* (%)	76.0		92.5		91.8	
Correlation** (%)	42.1		80.6		53.0	

*Sensitivity, specificity, positive predictive, negative predictive, and accuracy values were calculated according to total mycobacterial isolation.

**Pearson’s correlation.

NA, Not applicable.

## Discussion

Nucleic acid amplification tests have become popular for the rapid detection of *M. tuberculosis* in suspected TB patients. However, culture remains the gold standard method for the diagnosis of TB. Egg-based media such as Löwenstein-Jensen are widely used, but agar media such as Selective 7H11 and liquid-based media are now considered standard tools [7]. Several centers also use these systems to isolate rare mycobacterial species. Recently, TK SLC-L (Salubris, Inc.), a liquid version of the biphasic medium TK SLC, was introduced to the market. It enables the detection of *M. tuberculosis* complex (MTC) in clinical samples based on colourimetry. The culture system evaluated in this study incorporates several innovations, which make this new system convenient and feasible for microbiology laboratories.

The detection of *M. tuberculosis* in 146 specimens using TK SCL-L and MGIT produced similar results for 110 (75.3%) clinical specimens. A total of 23 (15.8%) cases were positive, and 87 (59.6%) cases were negative by both TK SCL-L and MGIT. However, discordance was obtained for 36 (24,7%) cases. TK SLC-L performed as well as MGIT in the isolation of mycobacteria from sputum samples. Although the difference was not statistically significant, TK SCL-L was superior to MGIT by isolating more mycobacteria among culture-positive specimens. These results are in agreement with the general acceptance of liquid culture systems as being superior to egg-based solid media [8].

In previous studies, the reported contamination rate for liquid culture systems was 10.0- 20.1%. TK SCL-L had a contamination rate of 1.3%, which is very low compared to that reported for other systems [2,9]. Although decontamination and concentration of the samples was done by the same technicians, and the same processed samples were inoculated into both TK SCL-L and MGIT, the contamination rate for MGIT (13.1%) was significantly higher than that for TK SCL-L (1.3%). This may be due to the pretreatments required for MGIT tubes. Rehydrating OADC and selective antimicrobials requires extra time and effort, and increases the risk of contamination. TK SLC-L liquid medium, which is used for the inoculation of clinical samples, contains selective antimicrobials to minimise contamination. All types of TK Media are ready-to-use; they do not require preparatory work prior to use. This eliminates the risk of contamination due to the extra manipulations required by other systems.

Sorlozano et al. (2009) compared MGIT, MB/BacT ALERT 3D, and LJ, and reported the time to growth detection as 15.1, 20.2, and 32.4 days, respectively [10]. In another study done by Saitoh and Yamane (2000), MGIT detected mycobacteria in 20 days, on average, compared to 17 days for MB/BacT [9]. In a multicentre study, the average time to growth detection for MGIT, BACTEC 460, and LJ was 13.3, 14.8, and 25.6 days, respectively [11]. Our average time to growth detection for MGIT (13.1 days) was comparable to previously published values. The average and median times to growth detection for TK SCL-L were 18.3 and 15.1 days, respectively. Although the time to growth detection was shorter for MGIT, there was no statistically significant difference between MGIT and TK SCL-L. However, the contamination rate was significantly higher for MGIT. The total time spent for the repetition of cultures for contaminated samples in MGIT make the total return time for culture results equal to or longer than the time required by TK SLC-L.

When the performance of TK SCL-L and MGIT were compared for the diagnosis of MTC, both systems did equally well in recovery and there were no differences in the sensitivity, specificity, positive predictive, negative predictive, and accuracy values. However, the correlation value between total culture positivity and MGIT positivity was less than that for TK SCL-L. This failure to recover mycobacteria by culture in some MGIT tubes was mostly due to the high contamination rate. In other studies, contamination rates between 2.0 and 20.1% have been reported for MGIT [3,9].

## Conclusions

By being ready to use and making the detection of mycobacterial growth and contamination easy (by following a colour change), TK SLC-L can be used efficiently for the diagnosis of TB in both elaborate and resource-limited laboratories. Having no requirement for media preparation before inoculation and a low contamination rate, TK SLC-L saves effort and time. (Table 2) Although, MYCOLOR TK, the automated system, makes it easy to follow a large number of culture tubes, the lack of requirement for an automated system is an important advantage of TK Media, enabling resource-limited laboratories to perform rapid mycobacterial culture. Future studies in different settings may be beneficial for further evaluation of the efficiency of TK SCL-L.

**Table 2:** Comparison of MYCOLOR TK and MGIT system

**Variables**	MYCOLOR TK*	MGIT*
Working principle	Colorimetric	Fluorometric
Sample capacity	336 to 2016 (modular)	960
Reading intervals	Adjustable (default set every two hours)	Once in a hour
Approximated test cost**	1.2-1.9 USD	4,5-7 USD
Approximated susceptibility and typing cost**	7.5-10.0 USD	18-22,5 USD
Shelf life	6-9 months	More than 1 year
Storage temperature for culture media	2-8°C	Room temperature
Storage temperature for supplements	2-8 C (Supplements included in TK media)	2-8 C
Online access from internet	Via epicenter	Via epicenter
*M. tuberculosis* – MOTT differentiation	Yes	Via extra test
Mycobacteria contamination differentiation	Via colorimetric or visual	Via turbidity or microscopy
Growth curves; auto-evaluation	Yes	Yes
Statistics	Yes	Via epicenter
Alternative visual evaluation without instrument	Yes	No

*Table was arranged according to manufacturer’s instructions.

**Approximated test cost, susceptibility and typing cost vary according to annual consumption amounts.

### Ethical approval

This was an experimental, randomized study that evaluated in vitro samples prospectively. No additional samples were taken from patients.

## Competing interests

The authors declare that they have no competing interests.

## Authors’ contributions

IHC designed the study, analyzed the results, reviewed the literature, prepared the manuscript, and contributed to drafting the manuscript. EK selected the samples, conducted the statistical analyses, and participated in the analysis of the results. Both authors read and approved the final manuscript.

## Pre-publication history

The pre-publication history for this paper can be accessed here:

http://www.biomedcentral.com/1471-2334/14/130/prepub
